# Ecological Risk Assessment and Protection Zone Identification for Linear Cultural Heritage: A Case Study of the Ming Great Wall

**DOI:** 10.3390/ijerph182111605

**Published:** 2021-11-04

**Authors:** Li Li, Rundong Feng, Jianchao Xi

**Affiliations:** 1 Key Laboratory of Regional Sustainable Development Modeling, Institute of Geographic Sciences and Natural Resources Research, Chinese Academy of Sciences, Beijing 100101, China; lil.20b@igsnrr.ac.cn (L.L.); fengrd.18s@igsnrr.ac.cn (R.F.); 2 College of Resources and Environment, Chinese Academy of Sciences, Beijing 100049, China

**Keywords:** ecological risk assessment, protection zoning, line-shape cultural heritage, the Ming Great Wall, driving mechanism

## Abstract

Ecological risk assessment is an important part of the sustainable development of World Heritage. The Ming Great Wall Heritage (MGWH) plays an important role in World Heritage conservation as a representative of large linear heritage, yet its ecological risks have not received much attention. This study assessed the ecological risk of MGWH based on simultaneous consideration of spatial heterogeneity and autocorrelation of geographic factors, and four protection zones were further identified from the perspective of preservation status and risk by using GeoDetector, principal component analysis and bivariate autocorrelation. The results showed that there were statistically significant differences in the preservation status of MGWH at different elevations. Based on this assessed ecological risk, it was found that 63.49% of MGWH grids were in the low to medium risk, while the highest risk areas (16.61%) were mainly concentrated in lower (200–500 m) and medium (500–1000 m) elevation. As elevation increased, the dominant factor of ecological risk shifted from human factors to natural factors and the main ecological risk showed a trend of increasing and then decreasing with increasing elevation. In addition, four types of risk protection zones (i.e., Protection—Restricted, Restoration—Moderate exploited, Restoration—Restricted and Protection—Moderate exploited) and policy suggestions were identified in this study from the perspectives of conservation, restoration and development, respectively. Future ecological protection of the MGWH should be based on the principle of “cultural heritage protection first”, with restricted development and use (e.g., tourism and education) and enhanced ecological restoration and environmental management of the surrounding area. This study provides references for the risk assessment of the cultural heritage at a large spatial scale, which is conducive to the maintenance and improvement of heritage value.

## 1. Introduction

World Heritage sites (WHS), which have Outstanding Universal Value (OUV) [1], are considered a critical enabler of sustainable development [2]. Target 11.4 of The 2030 Agenda for Sustainable Development proposes to protect and safeguard the world’s cultural and natural heritage [3]. Damage to any one of cultural heritage sites (CHSs) can endanger the livelihoods of local communities [4]. However, CHSs are facing catastrophic impacts, often causing irreversible damage [5]. The loss or deterioration of CHSs also has negative impacts on local and national communities, in terms of their intangible value as they represent icons of human civilization and symbols of identity, and their tangible value as they can bring benefits [6,7,8]. Therefore, it is necessary to establish a risk assessment framework for targeted risk responsive strategies and achieve the goal of sustainable development [9].

The Great Wall, which has been included in the World Heritage List by the United Nations Educational, Scientific, and Cultural Organization (UNESCO) since 1987, is an important frontier military system project in ancient China [10] and is the largest architectural heritage and military installation in the world [11]. However, the Great Wall has faced challenges against its sustainable development due to cracks, climate change, and human-related factors. For example, Li et al. [12] found that strong winds carrying sand and continuous rainfall form abrasion and erosion on the walls, which aggravate the cracks and collapses of the Great Wall sites. Meanwhile, rainfall and superimposed effects of soluble salts accelerate the cracking, bottom erosion, and collapse of the Great Wall sites [13]. Richards et al. [14] used the Vegetation and Sediment Transport model for the Heritage Deterioration model to assess the risk of environmental degradation in the Great Wall of Han Dynasty during a 100-year period and found that the increase in wind speed may greatly increase the overall deterioration. In addition, due to constructive and developmental destruction, the body of the Great Wall was severely damaged [15]. In order to obtain better protection, the State Administration of Cultural Heritage of the P.R. China launched a 10-year national Great Wall research and protection plan in 2005, called the Great Wall Protection Project (2005–2014) [16]. Since the launch of the project, great progress has been made in resource investigation, conservation and maintenance, fundamental management and legal system construction [16,17]. However, the Great Wall is currently not in very good condition and needs long-term protection measures [18,19]. It is always a great challenge to investigate the Great Wall heritage site since it covers huge areas [11,18], and sometimes data collection procedures might not be possible due to the lack of the appropriate equipment and tools [20]. Therefore, it is more practical to carry out a risk assessment based on some accessible, valuable information regarding natural and anthropogenic data and provide targeted strategies for protection.

Volumes have been written on sustainable development of CHSs, including policy-making, community involvement, social-economical influences, and risk assessment [21,22,23,24]. Among these, ecological risk assessment, as a crucial first step towards effective risk management and constituting the basis for taking preventive conservation measures for risk mitigation, is a growing topic [25,26]. Previous studies have shown that world heritage sites are threatened by both natural and anthropogenic threats [27]. Natural factors include elevation, soil erosion, landslides, floods, and rising sea levels. Elevation plays an important role in the risk of the CHS [28], which is mainly due to the redistribution of the hydrothermal environment and climate change [29]. In addition, 60% of the sites on the World Heritage List face at least one geological disaster, the most common of which are earthquakes and landslides [6]. For instance, the World Heritage Site in Kathmandu Valley of Nepal was damaged by a massive earthquake in 2015 [30], and the Bam Castle in Iran was damaged in 2003 [31]. Moreover, as the sea level rises, the risk of coastal disasters such as flooding and erosion increases [32]. A considerable number of world heritage sites in coastal areas will gradually be exposed to and be threatened by these hazards in the future [33]. Human factors, such as war [34], theft [35], tourism, and urbanization [36], are another major category of causes of the degradation and destruction of heritage sites. In particular, the land use changes caused by the expansion of agricultural land and urban development directly or indirectly affect the condition of the sites, thus destroying the historical resources [37,38].

However, most existing studies focus on the point-shape site groups and sites, and few involve line-shape cultural heritage [27,39,40]. Due to the large span and wide range of line-shape heritage, the areas it crosses may face different risks. If the risk value is calculated without considering regional heterogeneity, the result may be inconsistent with the local reality, which is not conducive to the targeted management of heritage risk. In addition, the preservation status of the sites is an important basis for the risk management strategy. Many studies have used heritage risk as a single reference criterion for decision making [20,41], while few articles have considered the conservation conditions of the site [8], which weakens the validity of the study results, because conservation measures of sites with different preservation conditions cannot be generalized in the same risk area. Therefore, this study takes the most representative Ming Great Wall heritage sites (MGWH) as an example to investigate the ecological risks via multiple methods, including GeoDetector, combining principal component analysis and spatial autocorrelation after identifying typical zoning elements and classifies the types of protection areas by comprehensively considering the preservation status. The results can provide specific suggestions for the sustainable development of the MGWH and guide future heritage conservation policies.

## 2. Materials and Methods

### 2.1. Study Area

The Great Wall was continuously constructed by more than 20 vassal states or dynasties for almost 2000 years and spreads over 15 provinces of China [42]. The Great Wall, especially the section built during the Ming dynasty, reflects collisions and exchanges between agricultural civilizations and nomadic civilizations in ancient China [16]. The Ming Great Wall is the most time-consuming, largest, and most well-developed defense system and structure in the history of China; it fully absorbs the characteristics of all dynasties and is also the best representative of the existing Great Wall heritages [11]. The MGWH therefore has irreplaceable value and status in the history of Chinese civilization and is considered the spiritual symbol of the Chinese nation.

**Figure 1:**
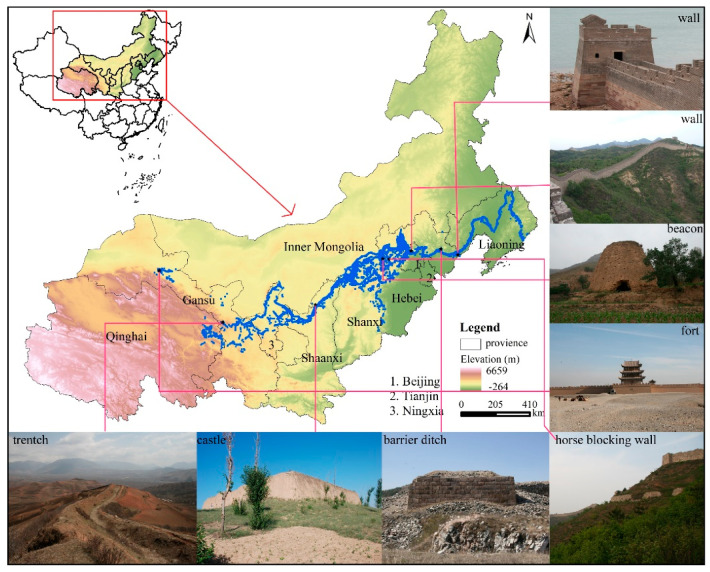
The Location of Ming Great Wall heritage sites (MGWH): more than 5200 sections of walls and trenches, more than 17,500 monolithic buildings, more than 1300 passages and castles, and more than 140 related facilities. Pictures from http://www.greatwallheritage.cn (accessed on 28 October 2021).

The MGWH starts from Tiger Mountain in Liaoning in the east and ends at Jiayuguan in Gansu in the west, with a total length of about 8852 km (Figure 1). It passes through 10 provinces, including Liaoning, Hebei, Beijing, Tianjin, Shanxi, Shaanxi, Inner Mongolia, Ningxia, Gansu, and Qinghai. More than 5200 sections of walls and trenches, more than 17,500 monolithic buildings, more than 1300 passages, and castles, and more than 140 related facilities exist. Its eastern section is mostly made of bricks, while the western section is mostly made of rammed earth. In addition, the MGWH can be found both in the warm temperate zone and the medium temperate zone, semi-humid and semi-arid zones, monsoon area and non-monsoon area, and low elevation (such as plain) and high elevation (including mountains, plateaus).

### 2.2. Influencing Factor Selection

To decide on the factors to include in the analysis, we conducted a three-week field survey and validation of the study area (MGWH) at different elevations through typical sample site selections, drone monitoring, and remote sensing. In addition, relevant experts involved in the conservation and development of the Great Wall were consulted, including various disciplines such as architecture, heritage conservation, and geography.

**Figure 2:**
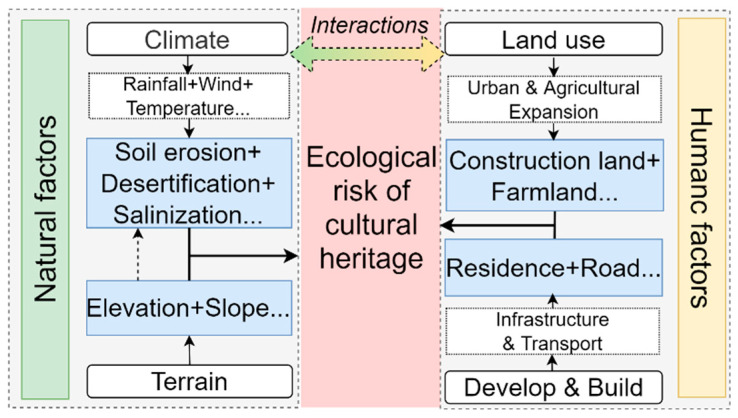
Interactions between natural and human factors and ecological risk framework of Ming Great Wall heritage sites.

By combining previous research [10,11], the main risk factors/threats identified by the UNESCO [43], and field advice from experts, we divided the risk factors of the MGWH into two categories: natural factors and human factors (Table 1 and Figure 2). Natural factors include rainfall, wind erosion, salt damage, and landslides, etc. [13]. Rainfall, wind erosion, soluble salt, and topography are considered as the main reasons for the deterioration of the MGWH [44,45]. However, rainfall data are time-variant, but the soil erosion data are effective indicators of rainfall erosivity because of their long-term and stable nature [46]. Wind and sand movements play an extremely important role in the formation and development of desertification [47], and the aeolian sand with sand grains contributes greatly to erosion on the MGWH, so aeolian sand erosion is used to represent desertification. In addition, the area with high soluble salt content in the soil is vulnerable to erosion and other degradation of the MGWH. Soil salinization is highly correlated with soil salinity [48], which can be used as an index of salt damage affecting the protection of the MGWH. Acid rain and landslides are important factors affecting heritage risks [20], as areas with higher slopes or frequent acid rain tend to damage the sites more easily. Therefore, natural factors such as soil erosion, desertification, salinization, acid rain erosion, and slope (Table 1) were considered in this study.

Human factors refer to the factors that lead to changes in the form of or in the conservation status of cultural heritage due to human activities [20], mainly including residents’ activities in the heritage sites, the expansion of surrounding cities, and land use changes. The MGWH was located in northern China, which is an important agricultural and pastoral ecotone area, and the human activities around it have an important influence on the degree of preservation of the sites [49,50]. In order to obtain more planting land, many areas directly bulldozed the site, so proximity to the cultivated land is often of great threat to the preservation of the site. In addition, the urbanization process caused by population growth, migration, and infrastructure development has a direct impact on cultural heritage sites. These impacts are positively correlated with the proximity to construction sites [36]. The accessibility of the existing road network to the site can promote future urban expansion and has a negative impact on the protection of cultural heritage sites. Air pollution near the highway often exceeds the normal limit and slowly damages the cultural heritage [20]. In addition, the residential area is also one of the important influencing factors. The closer to the residences, the more vulnerable it is to the damage of residents’ production and living activities [51]. Therefore, we selected the distance from cultivated land, construction land, road, and residences as human factors (Table 1).

### 2.3. Data Collection 

Datasets involved in this study mainly include the essential information of the MGWH and the risk factors that affect the preservation of the MGWH. We obtained essential MGWH information from the Great Wall heritage of China (http://www.greatwallheritage.cn/CCMCMS/) (accessed on 6 April 2021), which is based on a survey conducted by the State Administration of Cultural Heritage of China from 2007 to 2010. The essential information includes names, locations, materials, and preservation status of MGWH sections. The natural factors, such as soil erosion intensity (SEI), desertification intensity (DFI), salinization intensity (SAI), and acid-rain corrosion intensity (ACI), were obtained from the China Ecosystem Assessment and Ecological Security Database (http://www.ecosystem.csdb.cn/ecosys/index.jsp. (accessed on 6 April 2021), and the slope and elevation data were obtained from the 30-m resolution Digital Elevation Model (DEM) data of the Geospatial Data Cloud (http://www.gscloud.cn/) (accessed on 6 April 2021). The human factors such as distance to farmland (DisFL), distance to construction land (DisCL) and distance to road (DisRD) were calculated based on land use data of Resource and Environment Science and Data Center (https://www.resdc.cn/) (accessed on 6 April 2021), while the data of residences were obtained from Ministry of Civil Affairs (http://www.mca.gov.cn/article/sj/xzqh/1980/) (accessed on 6 April 2021).

### 2.4. Methodology

**Figure 3:**
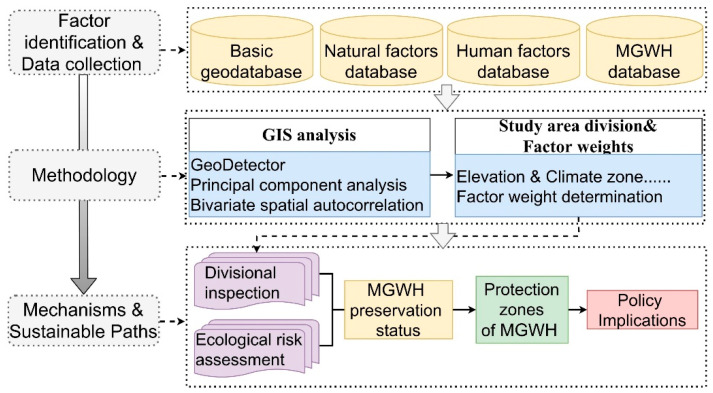
Methodological framework adopted in the ecological risk assessment and protection zone identification of the Ming Great Wall Heritage.

This paper is divided into three main steps (Figure 3). First, four databases on the ecological risk assessment of MGWH were established: basic database, natural factor database, human factor database, MGWH database. Then, the study area was divided by combining geospatial analysis and statistical analysis methods, and the weights of the influence factors were determined. Finally, the ecological risk of MGWH was evaluated, and the preservation degree was combined to obtain further conservation zoning and policy implications.

#### 2.4.1. GeoDetector

GeoDetector is a set of statistical methods for detecting spatial variability and revealing the driving forces behind it. The core idea assumes that if an independent variable has a significant effect on the dependent variable, then the spatial distribution of the independent variable and the dependent variable should be similar [52,53]. GeoDetector includes factor detection, interaction detection, risk area detection, and ecological detection. Risk area detection is used to determine whether there is a significant difference in the mean value of the preservation status of MGWH between two subregions. A t-statistic was used to test the reliability of risk area detection based on terrain partition in this study. The formula is as follows:

(1) $$t_{{\bar{y}}_{h=1}\;-\;{\bar{y}}_{h=2}\;=\;\frac{{\bar{Y}}_{h=1}\;-\;{\bar{Y}}_{h=2}}{{\left[\frac{Var\left({\bar{Y}}_{h=1}\right)}{n_{h=1}}\;+\;\frac{Var\left({\bar{Y}}_{h=2}\right)}{n_{h=2}}\right]}^{1/2}}}$$

where *Y_h_* represents the mean value of attributes in the subregion *h*; *n_h_* is the number of samples in the subregion *h*, and *Var* is the variance. The statistic *t* approximately obeys the Student’s *t* distribution. The degrees of freedom (the maximum number of logically independent values in samples) can be calculated as follows:

(2) $$df=\frac{\frac{Var\left({\bar{Y}}_{h=1}\right)}{n_{h=1}}+\frac{Var\left({\bar{Y}}_{h=2}\right)}{n_{h=2}}}{\frac{1}{n_{h=1}-1}[\frac{Var\left({\bar{Y}}_{h=1}\right)}{n_{h=1}}{]}^{2}+\frac{1}{n_{h=2}-1}[\frac{Var\left({\bar{Y}}_{h=2}\right)}{n_{h=2}}{]}^{2}}$$

The null hypothesis is *H*_0_: *Y_h_*_=1_ = ${\bar{Y}}_{h=2}$. If *H*_0_ is rejected at the confidence level *α*, it is considered that there is a significant difference in the mean value of the attributes between the two subregions.

#### 2.4.2. Principal Component Analysis

Principal component analysis (PCA) is a multivariate robust nonparametric approach that efficiently reduces the data dimension and identifies combinations of characteristics describing multivariate samples with the newly created principal components (PCs) [54,55]. It is a widely-used method, and some researchers have previously presented detailed information on PCA [56,57,58]. The objective of PCA is to extract the primary combination of information that is representative of the typical characteristics of the heritage risk. The basic procedures of the method are given in Supplementary Material.

The data were analyzed using SPSS 21. The Kaiser–Meyer–Olkin (KMO) test identifies whether the data are appropriate for PCA, and typically, a KMO sampling adequacy above 0.6 is acceptable [59]. The collected data received a 0.76 adequacy, indicating that the set of data is appropriate for PCA.

#### 2.4.3. Bivariate Spatial Autocorrelation

In this study, bivariate spatial autocorrelation is used to measure the overall trend and local spatial heterogeneity of the preservation status and the risk of the MGWH. Bivariate spatial autocorrelation comes in two types: global and local. The bivariate global spatial correlation is an indicator that describes the overall spatial relationship among all geographical units in the entire study area and can be calculated by the following formula:

(3) $$I=\frac{\sum_{i}^{n}\sum_{j\neq 1}^{n}w_{ij}\left(X_{i}-\bar{X_{i}}\right)\left(X_{j}-\bar{X_{j}}\right)}{S^{2}\sum_{i}^{n}\sum_{j\neq 1}^{n}W_{ij}}$$

where *I* is the bivariate global spatial correlation index (bivariate Moran’s *I*), *n* is the total number of grids, *W_ij_* is the spatial weight matrix, *X_i_* is the standard value of the preservation status of the site of grid *i*, and $\bar{X_{j}}$ is the standardized value of the risk degree of the site of grid *j.* The global Moran’s *I* value ranges from −1 to 1. When *I* is a zero, it represents space independence. When *I* > 0, it indicates a positive spatial correlation between preservation status and risk. The higher the value, the stronger the correlation. If *I* < 0, there is a negative spatial correlation. The statistical significance of Moran’s *I* can be tested using a permutation test. In this paper, statistical inference was based on 999 permutations [60], and the results of the statistical test can be observed in Table 2.

Bivariate local spatial autocorrelation mainly describes the spatial correlation within different spatial units, and the calculation formula is as follows [61]:

(4) $$I_{i}=z_{i}\overset{n}{\underset{j=1}{\sum }}w_{ij}z_{j}$$

In the formula, *I_i_* is the local correlation between a type of the MGWH condition in region *i* and the site risk in region *j*. There are mainly four clustering types, namely H-H, L-L, H-L, and L-H. *Z_i_* and *Z_j_* represent the variance standardized values of observed values of different variables in region *i* and *j*.

## 3. Results

### 3.1. Elevation Partition Test and Factor Weights

The MGWH crosses multiple elevation regions from east to west (Figure 1), and the influence of each index of its ecological risk at different elevations varies greatly [27]. Therefore, it is assumed that the preservation of the MGWH is greatly affected at different elevations. To verify the reliability of this hypothesis, the research area was divided into four topographic regions: lowest elevation region (0–200 m) (LTR), lower elevation region (200–500 m) (LRR), medium elevation region (50–1000 m) (MER), and high elevation region (>1000 m) (HER) according to the actual investigation. The study area was divided into 5 × 5 km risk assessment units, with a total of 2687 grids, to express the spatial regional heterogeneity of heritage risk. GeoDetector was used to verify the partition reliability.

Risk detectors were used to evaluate the reliability of the zoning based on elevation. We found that the degree of preservation of the MGWH was significantly different between elevation divisions except for between lower and medium elevation zones (Table 2). It is therefore reasonable to take elevation as the zoning basis for the ecological risk assessment of the MGWH. Based on this, PCA was used to determine the weight of each index according to the elevation partition. The PCA results are shown in Table 3. In general, in LRR, ecological risk was more influenced by anthropogenic factors, and with the increase in elevation, the dominant factors of ecological risk gradually shifted from anthropogenic factors (DisFL and DisCL) to natural factors (SEI, ACI, and Sp). Specifically, in LTR, ecological risk was mainly affected by the DisFL, Sp, and DisCL. The risk of MGWH in LRR was mainly affected by SEI, DisRS, and DisCL. Natural factors, such as SAI, DFI, ACI, and SEI, were the main factors affecting the ecological risk in MER, while human factors were less important. Sp, ACI, and DisCL were the leading factors of site risk in HER.

### 3.2. Ecological Risk of MGWH

**Figure 4:**
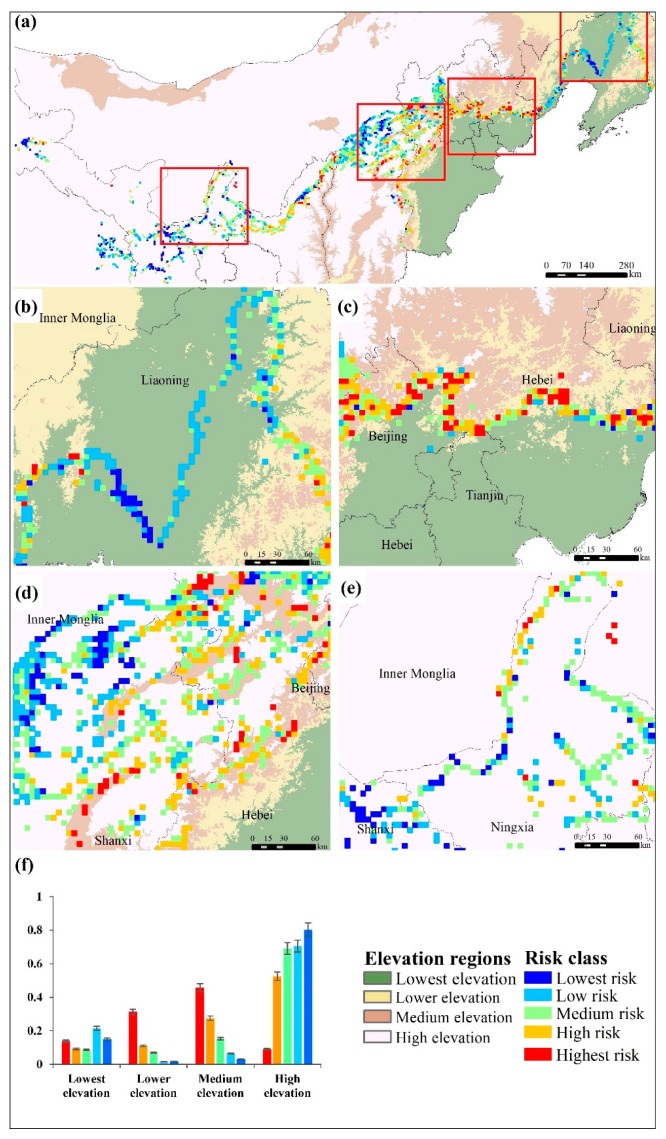
(**a**) Spatial distribution of the ecological risk in the Ming Great Wall heritage sites: (**a**) in all of the Ming Great Wall extension, (**b**) lowest elevation region, (**c**) lower elevation region, (**d**) medium elevation region, and (**e**) high elevation region, and (**f**) changes in the proportion of ecological risk levels with elevation.

Based on Table 3, the ecological risk of the MGWH was grouped into five categories (highest risk, high risk, medium risk, low risk, and lowest risk) according to the natural discontinuities. The risk level of the MGWH was mostly at the medium-low risk level (Figure 4a), with the highest proportion of medium risk grids (37.03%), followed by low risk grids (26.46%), and relatively few high risk (7.00%), lowest risk (10.9%) and highest risk (16.61%) grids. The medium risk level grids are mainly distributed in the central and western study region, which include Hebei, Shanxi, Shaanxi and Ningxia, and the low risk area is mostly located in the eastern and central regions such as Liaoning and Shanxi. In addition, highest and high risk grids were concentrated in central regions such as Hebei and Shanxi, while the lowest risk grids were mainly found in the eastern and western regions, which include Liaoning, Gansu and Qinghai. Therefore, with the gradually increasing elevation, the risk of the MGWH presented a distribution of “medium–low risk—medium risk–low risk”.

In LTR, the risk of the MGWH was dominated by low-risk areas (43.09%) and medium-risk areas (24.23%) (Figure 4f). The low-risk areas are mainly located in Liaoning (Figure 4b) and have good spatial continuity. The high-risk areas are mainly located in areas adjacent to hills in Beijing, Tianjin, and Hebei. The MGWH in LRR had the highest overall risk, which was dominated by medium-risk areas (34.67%), highest-risk areas (29.65%), and high-risk areas (27.64%) (Figure 4f), and the three types of grids in LRR were mainly concentrated on the west of Beijing and the border of Hebei (Figure 4c). The low-risk areas were mostly located in the eastern and western Liaoning and the eastern part of Hebei. In MER, the areas with medium risk (35.50%) and high risk (31.79%) accounted for a large proportion (Figure 4f), which are clustered in the mountainous areas of Beijing, Hebei, and Shanxi (Figure 4d), while the lower-risk areas were mainly located in the western part of Hebei. In HER, the proportions of medium-risk, lowest-risk, and low-risk grids were 40.36%, 29.44%, and 13.81%, respectively (Figure 4f). The medium-risk and lowest-risk grids were mainly concentrated in Ningxia, Qinghai and northwestern Gansu (Figure 4e). With the increase in overall risk, the proportion of the MGWH in LTR significantly increased first and then decreased, while the proportion of sites in LRR and MER rapidly increased by 29.84% and 42.67%, respectively. Medium-risk and high-risk areas were always transitional zones.

### 3.3. Protection Zones of MGWH

**Figure 5:**
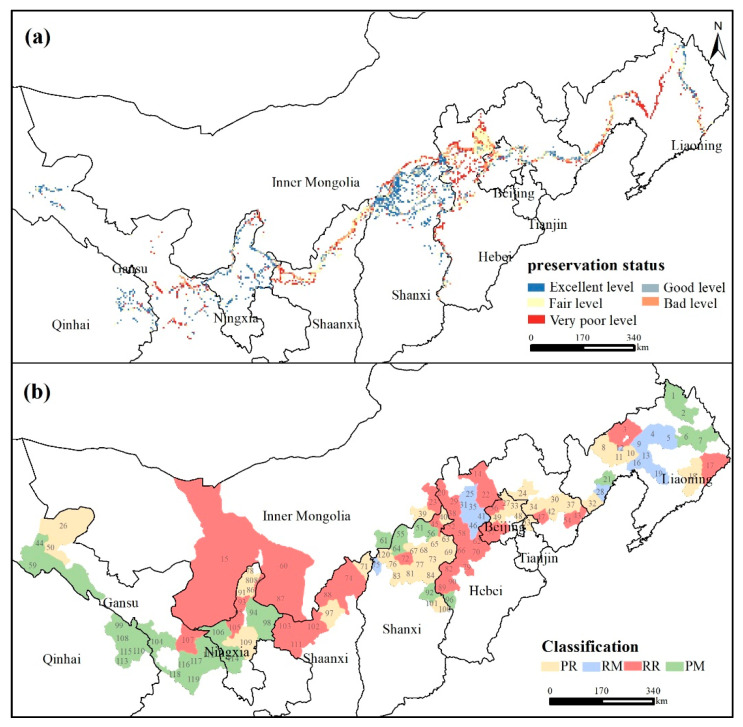
Preservation status of the Ming Great Wall Heritage: (**a**) different degrees of preservation status, (**b**) protection zones at the county level. PR: Protection—Restricted zone; RM: Restoration—Moderate exploited zone; RR: Restoration—Restricted zone; and PM: Protection—Moderate exploited zone.

Risk assessment is important to protection zoning, while the preservation status can be different even under the same risk level. Therefore, it is necessary to comprehensively consider the risk and preservation status of the sites to form a protection zone. As shown in Figure 5a, the preservation status of the MGWH is mostly at the excellent (27.24%) and fair levels (25.27%), which are distributed in Hebei, Shanxi and Ningxia. The grids with very bad levels are also in great numbers (22.11%), which concentrate in Liaoning, central Hebei, western Shaanxi and eastern Gansu. Based on the global bivariate Moran’s *I* analysis, we found that the preservation status of the MGWH under the four elevation types presented different spatial correlations with the risks (Table 4). In particular, the global bivariate Moran’s *I* value of low elevation (*p* < 0.05) and medium elevation (*p* < 0.05) was greater than 0, indicating that there is a positive spatial correlation between the preservation status of the MGWH and the risk. However, the global bivariate Moran’s *I* value was less than 0 at lower (*p* < 0.1) and high (*p* < 0.01) elevations, indicating a negative spatial correlation between preservation status and risk.

Therefore, we used bivariate local spatial autocorrelation to identify four types of risk management zones for the MGWH (Table 4). The first type is Protection—Restricted zone (PR), which represents good-conservation sites surrounded by high-risk areas. The second type is Restoration—Moderate exploited zone (RM), which represents poor-conservation sites surrounded by low-risk areas. The third type is Restoration—Restricted zone (RR), which represents poor-conservation sites surrounded by high-risk areas. The fourth type is the Protection—Moderate exploited zone (PM), which represents good-conservation sites surrounded by low-risk areas. To make the results more operational and increase the practical guiding significance, the results of risk management zoning were summarized to the county-level districts in a statistical manner based on the proportion of the county-level districts (Table S1 in the Supplementary Material and Figure 5b). If the proportion of multiple types is equal, the cultural heritage protection should be the first principle, and it can be summarized as the RR type. Overall, PR and RM were dominant in LTR and MER, while RR and PM were dominant in LRR. RR and RM accounted for the largest proportion in HER. Specifically, the PR type was mainly concentrated in the Beijing-Tianjin-Hebei urban agglomeration (e.g., Kuancheng, Ji, and Huairou) and northwestern Shanxi (e.g., Guangling, Lingqiu, and Wutai), showing a typical “cluster pattern”. RM type was mainly concentrated in central Liaoning (e.g., Heishan, Tai ‘an, and Panshan) and central Hebei (e.g., Chongli, Xuanhua, and Zhuolu), and its spatial distribution was relatively concentrated. RR type was mainly distributed in “U” type areas with Shangyi, Yixian, and Chicheng as nodes and the line of Alashan Left Banner–Etoke Banner–Dingbian–Jingbian, with obvious spatial continuity. The PM type showed line-shape distribution with Yugu Autonomous in Sunan, Menyuan Hui, Tianzhu Tibetan, and Yuzhong-Jingyuan-Zhongwei as nodes.

## 4. Discussion and Implications

### 4.1. Discussion

**Figure 6:**
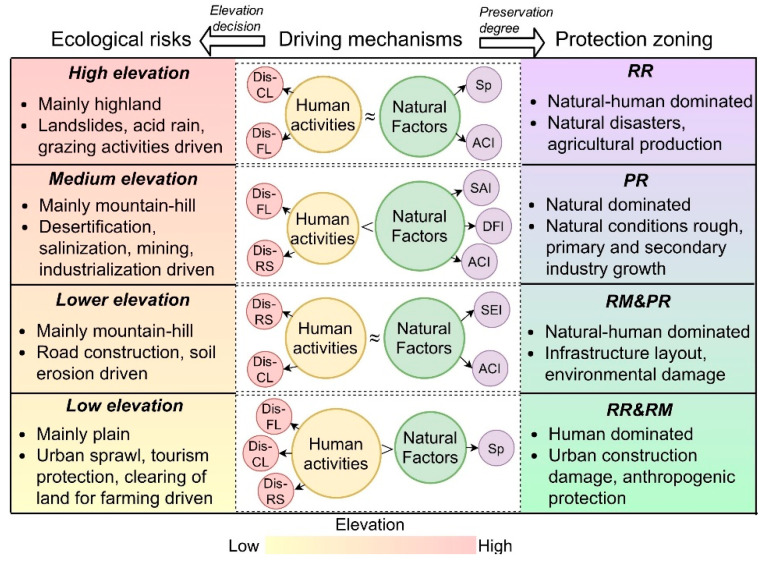
Driving mechanisms for ecological risk assessment and protection zoning of the Ming Great Wall Heritage sites. Abbreviations of influence factors are shown in Table 1.

With an increasing elevation, the primary ecological risk of the MGWH changes from anthropogenic factors to natural factors (Figure 6). The overall ecological risk, meanwhile, increases first and then decreases. This pattern is mainly due to the elevation-driven reallocation of the hydrothermal conditions [28] and the elevation-related difference of geomorphic types and climate characteristics [58], which both affect the population agglomeration pattern and production and construction activities [29]. For example, the medium and low elevation regions have gentle terrain and good water and heat conditions [62], so a large number of people are concentrated in these areas, and the intensity of agricultural development and urban construction (such as urban land expansion and road construction) is high. Therefore, the ecological risk of the MGWH is greatly influenced by anthropogenic factors such as DisCL, DisRS, and DisRD. Reversely, SAI, ACI, and Sp are dominant risk factors in the medium and high elevation regions, where the population is sparsely populated and climate change (e.g., rainfall and temperature) and natural disasters (e.g., salinization, soil erosion, and landslides) significantly control ecological risks [63,64]. In addition to elevation, other factors also have an important influence on the preservation state of MGWH, such as climate. However, since the climate is formed by the interaction of many factors, the climate factors are changeable and the influence mechanism is complex, so it is difficult to use a single factor to characterize its influence on the cultural heritage [65]. Therefore, this study used GeoDetector to identify terrain as the basic geographical element of ecological risk of the Ming Great Wall in Dynasty, which is also consistent with the research results of Agapiou [20].

The sheer extent of the MGWH and the intricate topography through which it passes led to significant differences in the driving mechanisms of ecological risk at different elevations (Figure 6). For example, the main source of ecological risk in the LTR was infrastructure construction such as tourism and agricultural activities [66], so ecological risks are highly correlated with DISFL. In addition, the high population density, urban expansion, and road network construction in LTR have strengthened the ecological threat of DisRD and DisRS to the MGWH. However, the overall ecological risk in LRR was influenced by a combination of anthropogenic and natural factors. This is due to the fact that LRR was mostly located in the monsoon region of east-central China [15], and the predominantly hilly terrain led to severe erosion and natural disasters such as landslides [58,67], making MGWH severely damaged [68]. Furthermore, a widespread conversion from farmland to forest reduced agricultural planting activities [69], but the impact of human production and construction was still significant, which together with natural factors control the ecological risk. MER is the area most affected by sandstorms in China [70], and unreasonable reclamation practices and other natural disasters have led to desertification [71], mudslides and other natural disasters [72]. Therefore, medium-high risk dominated by natural factors were the main types of MER. Finally, HER was rich in mineral resources [73] and passed through areas of acid rain concentration (e.g., Hebei, Shanxi) and areas of severe salinization (e.g., Ningxia) [74], thus both natural and human factors seriously threatened the preservation of MGWH.

Traditionally, many protection zones are simply identified based on their ecological risk, especially for point heritage sites [33]. However, this partition method has the problem of one-size-fits-all because it does not consider the status quo of the site. This study finds that for linear cultural heritage at large spatial scales, it is more practical to combine the regional autocorrelation of ecological risk and preservation status for the identification of protection zones based on the spatial heterogeneity of ecological risk. For example, in LTR dominated by plains, many MGWH have been built as tourist attractions due to the development of tourism [24], and combined with the enforcement of heritage conservation, PR has become the dominant type. Nevertheless, RM is also scattered in LTR (such as Heishan, Haicheng), due to past wars [75], earthquakes [76], etc. In LER, the terrain is dominated by plains and hills, and because of the dense population, some of the roads and dwellings are even built directly on the MGWH [67,77], leading to the RR as the main protection type. With the increase in elevation, the MER, where hills and mountains are the main terrain, has the poorly preservation of MGWH due to the debris flow [58], acid rain [78], and salinization [79]. However, some parts of the MGWH are combined with the natural terrain to form mountain risks (e.g., Huairou, Xinglong, and Yuanping) during the construction, and the structures are relatively stable, so the distribution of the four protection types in MER was relatively even. In HER, due to the large proportion of highlands and mountains, the surrounding areas of the MGWH are important agricultural and pastoral areas and mineral resource areas [15,80,81], in addition to natural hazards such as salinization and desertification, agricultural and mining activities have also had a significant impact on the site. Therefore, in the area with strong agricultural development and more natural disasters such as Pingluo, Hengshan, and Xuanhua, “restoration type” (RR and RM) was the main type. However, in the area dominated by natural factors such as Eketuo, Dingbian, and Jingyuan, PM and PR were dominant.

### 4.2. Policy Implications

The preservation status of the MGWH is closely related to the risk of where it is located. It is necessary to consider the risk and preservation as a whole and put forward reasonable suggestions (Table 5). Specifically, for PR type areas, the risk is high, but the preservation condition of the site is good. These areas need to protect the cultural heritage sites. Avoiding the cause of risks and everything that makes risks higher is the priority. Second is detecting the agents of deterioration and their effects on the site. Then, effective responses are needed whenever a problem is detected. At the same time, in view of the cultural importance of the MGWH, the well-preserved areas can be used for restricted development and utilization within the carrying capacity, mainly for the construction of sightseeing facilities. In addition, appropriate path design will minimize the impact of visitors. For RM areas, the preservation status of the sites is poor, but the risk levels are low. Due to the small influence of the surrounding environment, the restoration of the sites in this kind of protection area is the first logical action. At the same time, potential risk-detection and routine maintenance of sites should be implemented to prevent the ruins from worsening. Given the development demand of local communities, only authorized livelihood activities are permitted. In addition, the local community can and should play an important role in the protection of the site. Therefore, we need to develop tools and learning resources based on the careful consideration of user needs and knowledge gaps [82], which will help raise their awareness of protection. In RR areas, the site condition is poor and the risk levels are high. Therefore, the primary purpose of this kind of protection area is to restore the damaged sites to the maximum extent. At the same time, close attention should be paid to the high-risk factors in this area, such as soil erosion, acid rain erosion, landslides, and salinization factors, and the environmental management of the surrounding area should be strengthened. In addition, local community participation should be involved. PM type areas are in good site condition with low ecological risk, which can be used as the main window for publicizing and displaying the MGWH. Though these areas are in low risk levels, it is necessary to monitor the potential risks so that we can react quickly in case they threaten the heritage asset. Another action is forming a reasonable visitor management strategy, including determining the carrying capacity of sites, designing paths for visitors within the site, creating a buffer zone and equipping the sites with the facilities providing various services in appropriate areas.

### 4.3. Innovation and Limitations

Ecological risk assessment of cultural heritage at the landscape scale has proven to be more cost-effective and efficient than traditional methods based on large-scale observation and monitoring. In addition, due to the large span of the MGWH, the local dominant factors need to be adjusted. In this study, based on different elevation types, the analytic hierarchy process is respectively used to assign the weight of the risk index, which improves the accuracy of the calculation of the risk of the MGWH to a certain extent, provides relevant reference for the protection of large-scale line-shape sites, and contributes to the management of cultural resources.

However, there are some limitations to note. First, the selection of factors in this study was incomplete due to limitations in data availability, although representative drivers were chosen. Second, we focused only on the absolute values of these weights, but their statistical significance was not clarified, which may have affected model accuracy. Hence, decision makers should pay more attention to these factors, such as periodic state recording and assessment. Especially for the factors that can change dynamically (e.g., rainfall, land use, and vegetation index), it is necessary to monitor their potential changes. Thirdly, incomplete information and data, criteria layers and risk values are inevitably overestimated or underestimated in the risk assessment [8]. Therefore, it is important to know the degree of uncertainty of each of the results [9].

## 5. Conclusions

China has issued a plan on the construction of national culture parks for the Great Wall and aims to complete the construction of the parks by 2023 with related cultural resources enjoying better protection, inheritance and utilization. Considering the increasing need for risk evaluation that supports informed and balanced decision-making, the estimation of risk is crucial for the effective risk management of CHS by providing better protection targets. This study, by using remote sensing image data, vector data, and the risk project, based on GeoDetector, PCA, and bivariate spatial autocorrelation, respectively, partitions the MGWH into elevation areas and then evaluates the regional ecological risk from the perspective of site risk and preservation conditions, and finally proposes four types of risk management and puts forward related suggestions. The results show that with the increase in elevation, the major risk of the site changed from human factors to natural factors. Spatially, the low- and medium-risk areas were mainly concentrated in LTR and HER, while the medium- and high-risk areas were mainly concentrated in LRR and MER. Since all risks in this article are identified and mapped through remote sensing data and geographic big data, the proposed method can be easily applied to any other area of interest to assess the overall risk of different monuments and sites and can support adaptation planning for the conservation of cultural heritage with a large span scale such as the Great Wall. In the future, more detailed data and level of (un)certainty will be combined to strengthen further discussion of typical areas in different regions, and risk management strategies at different research scales will be comprehensively considered so as to provide more effective guidance for the protection of the MGWH.

## Figures and Tables

**Table 1:** Factors, indicators and data sources considered in the Ming Great Wall Heritage sites risk evaluation.

Factors	Indicators	Resources	Explanation of Indicators	Abbreviation	Resolution
Natural factors	Soil erosion intensity	http://www.ecosystem.csdb.cn/ecosys/index.jsp (accessed on 6 April 2021)	Divided into five types of risk levels: general, mild, moderate, high, and extremely sensitive	SEI	1 km
	Desertification intensity			DFI	1 km
	Salinization intensity			SAI	1 km
	Acid-rain corrosion intensity			ACI	1 km
	Slope	http://www.gscloud.cn/ (accessed on 6 April 2021)	Calculated from the elevation	Sp	1 km
Human factors	Distance to farmland	https://www.resdc.cn/ (accessed on 6 April 2021)	Euclidean distance to farmland	DisFL	1 km
	Distance to construction land		Euclidean distance to construction land	DisCL	1 km
	Distance to road		Euclidean distance to road	DisRD	1 km
	Distance to residences	http://www.mca.gov.cn/article/sj/xzqh/1980/ (accessed on 6 April 2021)	Euclidean distance to residents	DisRS	1 km

**Table 2:** Four types of elevation regions of the Ming Great Wall Heritage and degree of preservation statistic differences as a support for risk detection.

	LTR	LRR	MER
LRR	Y		
MER	Y	Y	
HER	Y	N	Y

Y denotes a statistically significant difference between elevations; N denotes no statistically significant differences between elevations. LTR: lowest elevation region, LRR: lower elevation region, MER: medium elevation region, and HER: high elevation region.

**Table 3:** Principal component analysis (PCA) results in the four elevation regions of Ming Great Wall Heritage (%).

	LTR	LRR	MER	LTR
SEI	8.52	27.06	15.16	9.34
DFI	11.09	4.26	16.26	9.41
SAI	2.66	1.82	17.91	12.92
ACI	6.65	13.9	15.95	14.72
Sp	15.42	6.22	12.34	16.66
DisFL	17.77	4.76	11.63	13.32
DisCL	12.96	15.44	1.81	14.11
DisRD	12.19	9.76	2.83	7.65
DisRS	12.74	16.78	6.11	1.87

LTR: lowest elevation region, LRR: lower elevation region, MER: medium elevation region, and HER: high elevation region. Abbreviations of influence factors are shown in Table 1.

**Table 4:** Bivariate Moran’s *I* and local spatial autocorrelation between preservation status and ecological risk in the Ming Great Wall Heritage.

Type	Bivariate Moran’s *I*	Protection-Restricted (PR)	Restoration-Moderate Exploited (RM)	Restoration-Restricted (RR)	Protection-Moderate Exploited (PM)
LTR	0.31 ***	43.30%	37.11%	12.37%	7.22%
LRR	−0.04 *	15.63%	15.63%	37.50%	31.25%
MER	0.05 **	27.14%	26.09%	23.91%	22.83%
HER	−0.04 ***	20.65%	25.15%	29.86%	24.34%

*** denotes statistical significance at the 1% level; ** denotes statistical significance at the 5% level; * denotes statistical significance at the 10% level. LTR: lowest elevation region, LRR: lower elevation region, MER: medium elevation region, and HER: high elevation region.

**Table 5:** Proposals of protection zone types for the Ming Great Wall Heritage sites.

Risk Management Type	Characteristics	Typical Districts
Protection—Restricted zone (PR)	Condition: high risk and good preservation Actions: (1) avoid the cause of risks and everything that makes risks higher; (2) detect the agents of deterioration and their effects on the site; (3) determine the carrying capacity of sites and design paths for visitors within the site.	Beizhengmanzuzizhi, Yi, Kuancheng, Ji, Pinggu, Huairou, Luanping, Xinglong.
Restoration—Moderate exploited zone (RM)	Condition: low risk and poor preservation. Actions: (1) detect the potential risks and avoid everything that makes risks higher; (2) recover the items or parts of the affected heritage asset; (3) discourage unauthorized activities in vulnerable areas of heritage sites; (4) involve local communities in conservation.	Chongli, Xuanhu, Zhuolu, Hequ, Huailai, Xincheng, Taian, Panshan.
Restoration—Restricted zone (RR)	Condition: high risk and poor preservation. Actions: (1) avoid the cause of risks and everything that makes risks higher; (2) detect the agents of deterioration and their effects on the site; (3) recover the damaged sites; (4) involve local communities in conservation.	Fangshan, Yanqing, Chicheng, Alashanzuoqi, Zhongning, Dingbian, Jingbian, Shengmu.
Protection—Moderate exploited zone (PM)	Condition: low risk and good preservation. Actions: (1) detect the potential risks and avoid everything that makes risks higher; (2) determine the carrying capacity of sites and design paths for visitors within the site; (3) create a buffer zone and equip the sites with the facilities providing various services.	Changtu, Funing, Fushun, Lianshan, Jingjin, Meng, Gaolan, Yuzhong.

## Data Availability

Not applicable.

## Supplementary Materials

The following are available online at https://www.mdpi.com/article/10.3390/ijerph182111605/s1, Table S1: The name of the region that each ID represents.
