# Supplementary material for: Ecological Risk Assessment and Protection Zone Identification for Linear Cultural Heritage: A Case Study of the Ming Great Wall
Source: Int J Environ Res Public Health. 2021 Nov 4;18(21):11605. doi: 10.3390/ijerph182111605 (PMC8583037; doi:10.3390/ijerph182111605)
Supplement: Supplementary file 1 [file ijerph-18-11605-s001.zip › ijerph-1420056-supplementary.pdf]

Supplementary material

The procedures of principal component analysis (PCA) are as follows:

The initial dataset (*X*) can be represented in matrix form:

$$X = \begin{bmatrix} x_{11} & x_{11} & \cdots & x_{1m} \\ . & . & \cdots & . \\ . & . & \cdots & . \\ x_{n1} & x_{n1} & \cdots & x_{nm} \end{bmatrix} \tag{1}$$

where *m* is the number of causal factors, *n* is the landslide number, and x_{ij} (i=1, 2, …, n; j=1, 2, …, m) is the jth factor of the ith landslide. The mean and standard deviation of these factors can be calculated as follows:

$$\overline{x_j} = \frac{1}{n}\sum_{i=1}^{n} x_{ij} \tag{2}$$

$$S_j = \sqrt{\frac{1}{n-1}\sum_{i=1}^{n}(x_{ij} - \overline{x_j})^2} \quad (j=1, 2, ..., m) \tag{3}$$

where $\overline{x_j}$ and S_j are the mean and standard deviation of factor j, respectively. These two parameters can be used to normalize the original data *X* and obtain the correlation matrix $R = (r_{jk})_{m\times m}$:

$$y_{ij} = \frac{x_{ij} - \overline{x_j}}{S_j} \quad (i=1, 2, ..., n; j=1, 2, ..., m) \tag{4}$$

$$r_{jk} = \frac{1}{n-1}\sum_{i=1}^{n}\frac{(x_{ij} - \overline{x_j})}{S_j}\frac{(x_{ik} - \overline{x_k})}{S_k} \quad (j=1, 2, ..., m; k=1, 2, ..., m) \tag{5}$$

The eigenvalue and eigenvector of matrix *R* can be determined:

$$(R - \lambda_i I)l_i = 0 \tag{6}$$

where λ_i (i=1, 2, …, m) and l_i (i=1, 2, …, m) are the eigenvalues and eigenvectors of matrix *R*, respectively, l_i corresponds to the principal components, and λ_i corresponds to the variance obtained from each principal component. The effect of each eigenvalue is given by the contribution rate. A larger contribution rate indicates a larger eigenvalue. The largest eigenvalues represent the principal components regarding most of the variability in the observed data. The cumulative contribution rate α for a specific eigenvalue λ_k (i=1, 2, …, m) can be obtained as follows:

$$\alpha = \frac{\lambda_1 + \lambda_2 + ... + \lambda_k}{\lambda_1 + \lambda_2 + ... + \lambda_m} \times 100\% \tag{7}$$

If the value of α is equal to or more than 90%, *k* principal components are considered to contain sufficient information to represent the complex original data array. The matrix $(F_{ij})_{n\times k}$, composed of *k* principal components, can be expressed by:

$$F_{ij} = \sum_{i=1}^{m} y_{it} l_{tj} \quad (i=1, 2, ..., n; j=1, 2, ..., k; t=1, 2, ..., m) \tag{8}$$

In this matrix, the largest contribution rate is given by the first principal component, followed by the other components, which have gradually decreasing contribution rates.

Table S1: The name of the region that each ID represents

ID	NAME	ID	NAME
1	changtuxian	61	qingshuihexian
2	qinghe	62	laishuixian
3	fuxinmengguzuzizhixian	63	guanglingxian
4	xinminxian	64	pingluqu
5	shenbeixinqu	65	hunyuanxian
6	fushunxian	66	laiyuanxian
7	xinbinmanzuzizhixian	67	shanyinxian
8	beipiaoshi	68	yingxian
9	heishanxian	69	lingqiuxian
10	beizhenmanzuzizhixian	70	yixian
11	yixian	71	fuguxian
12	qinghemenqu	72	shouzhoushi
13	taianxian	73	fanshixian
14	guyuanxian	74	shenmuxian
15	alashanzuoqi	75	hequxian
16	panshanxian	76	shencixian
17	kuandianmanzuzizhixian	77	daixian
18	fenchengmanzuzizhixian	78	shizhuishanshi
19	haichengshi	79	tangxian
20	shangyixian	80	pingluoxian
21	lianshanqu	81	yuanpingshi
22	cichengxian	82	fupingxian
23	xinghexian	83	ningwuxian
24	luanpingxian	84	wutaixian
25	chonglixian	85	taolexian
26	jintaxian	86	helanxian
27	huairouxian	87	ertuokeqianqi
28	xingchengshi	88	yulinqu
29	wanquanxian	89	pingshanxian
30	kuanchengmanzuzizhixian	90	lingshouxian
31	xuanhuaqu	91	yinchuanshi
32	suizhongxian	92	mengxian
33	miyunxian	93	yongningxian
34	xinglongxian	94	lingwuxian
35	xuanhuaxian	95	qingtongxiashi
36	yanqingxian	96	jingjingxian
37	qinglongmanzuzizhixian	97	hengshanxian
38	huaianxian	98	yanchixian

39	fengzhenshi	99	menyuanhuizuzizhixian
40	tianzhenxian	100	pingdingxian
41	huailaixian	101	yangquanshi
42	qianxixian	102	jingbianxian
43	funingxian	103	dingbaixian
44	jiyuguanshi	104	tianzhuzhangzhuzizhixian
45	yanggaoxian	105	zhongningxian
46	zhuoluxian	106	zhongweixian
47	zhunhuashi	107	jingtaixian
48	pingguxian	108	datonghuizutuzuzizhixian
49	changpingxin	109	tongxinxian
50	jiuquanshi	110	huzhutuzuzizhixian
51	nanjiaoqu	111	wuqixian
52	yangyuanxian	112	jingyuanxian
53	jixian	113	huangzhongxian
54	lulongxian	114	haiyuanxian
55	youyuxian	115	chengzhonqu
56	datongxian	116	gaolanxian
57	mentougou	117	baiyinqu
58	weixian	118	honguqu
59	shunanyuguzuzizhixian	119	yuzhongxian
60	ertuokeqi	120	pianguanxian
